# Screening for Activity Against AMPA Receptors Among Anticonvulsants—Focus on Phenytoin

**DOI:** 10.3389/fphar.2021.775040

**Published:** 2021-12-07

**Authors:** M. Y. Dron, A. S. Zhigulin, D. B. Tikhonov, O. I. Barygin

**Affiliations:** I.M. Sechenov Institute of Evolutionary Physiology and Biochemistry RAS, Saint-Petersburg, Russia

**Keywords:** AMPA receptor, pharmacological modulation, patch–clamp technique, screening, anticonvulsants, phenytoin

## Abstract

The interest in AMPA receptors as a target for epilepsy treatment increased substantially after the approval of perampanel, a negative AMPA receptor allosteric antagonist, for the treatment of partial-onset seizures and generalized tonic-clonic seizures. Here we performed a screening for activity against native calcium-permeable AMPA receptors (CP-AMPARs) and calcium-impermeable AMPA receptors (CI-AMPARs) among different anticonvulsants using the whole-cell patch-clamp method on isolated Wistar rat brain neurons. Lamotrigine, topiramate, levetiracetam, felbamate, carbamazepine, tiagabin, vigabatrin, zonisamide, and gabapentin in 100-µM concentration were practically inactive against both major subtypes of AMPARs, while phenytoin reversibly inhibited them with IC50 of 30 ± 4 μM and 250 ± 60 µM for CI-AMPARs and CP-AMPARs, respectively. The action of phenytoin on CI-AMPARs was attenuated in experiments with high agonist concentrations, in the presence of cyclothiazide and at pH 9.0. Features of phenytoin action matched those of the CI-AMPARs pore blocker pentobarbital, being different from classical competitive inhibitors, negative allosteric inhibitors, and CP-AMPARs selective channel blockers. Close 3D similarity between phenytoin and pentobarbital also suggests a common binding site in the pore and mechanism of inhibition. The main target for phenytoin in the brain, which is believed to underlie its anticonvulsant properties, are voltage-gated sodium channels. Here we have shown for the first time that phenytoin inhibits CI-AMPARs with similar potency. Thus, AMPAR inhibition by phenytoin may contribute to its anticonvulsant properties as well as its side effects.

## Introduction

Epilepsies are among the most common chronic brain disorders (Scharfman, 2007). They affect 0.5–1% of people around the world (Sirven, 2015). Despite the constant development of new antiseizure drugs during the last decades (Rho and White, 2018), 20–30% patients cannot control seizures even with modern medications. Thus, the search for new anticonvulsant drugs and detailed understanding of the mechanisms of action of older ones are extremely important for effective selection of therapy for each patient. Seizures may produce neurodegeneration within the brain, and different antiseizure drugs have different potential to prevent it (Miziak et al., 2020).

According to a recent review by Sills and Rogawski (2020), there are four major classes of antiseizure drug mechanisms: 1) modulation of voltage-gated ion channels; 2) enhancement of GABA-mediated inhibitory neurotransmission; 3) attenuation of glutamate-mediated excitatory neurotransmission; and 4) modulation of neurotransmitter release via a presynaptic action. Combining two or more of these mechanisms in one drug can be beneficial for seizure control.

Approval of perampanel—a negative allosteric AMPA receptor antagonist—enhanced the interest in testing whether older antiseizure drugs can affect AMPA receptors (Fukushima et al., 2020). Indeed, several other anticonvulsants were shown to affect AMPA receptors. Lamotrigine inhibited postsynaptic AMPA receptors and glutamate release in the dentate gyrus (Lee et al., 2008); however, 30 and 100 µM lamotrigine decreased the amplitude of the currents induced by exogenously applied AMPA by 10% only (Lee et al., 2008). Topiramate concentrations of 30 and 100 µM inhibited AMPA and kainate-evoked Ca^2+^ uptake in cultured cerebral cortical, hippocampal, and cerebellar neurons by up to 60% (Poulsen et al., 2004). But the effect of topiramate on AMPA receptors might be indirect (Gibbs et al., 2000; Angehagen et al., 2004). Levetiracetam (200 µM) decreased the amplitude of kainate-induced current in cultured cortical neurons by about 26% (Carunchio et al., 2007). Finally, phenytoin inhibited non-NMDA glutamate receptors expressed in *Xenopus* oocytes (Kawano et al., 1994) and in neocortical wedges (Phillips et al., 1997) with IC50 values ≥100 µM. These data attract attention to AMPA receptors as a potential target for different antiseizure drugs, but it is not clear whether AMPA receptor inhibition can contribute to their therapeutic and side effects. Thus, we decided to perform a broad screening for activity against AMPARs among these and some other antiepileptic agents.

Two major subtypes of AMPARs—calcium-permeable (CP-AMPARs) and calcium-impermeable (CI-AMPARs)—play different roles in maintaining the excitation–inhibition balance in the brain. CP-AMPARs are usually localized in GABA-ergic interneurons, whereas principal cells in many brain structures contain CI-AMPARs (Buldakova et al., 1999; Samoilova et al., 1999). Selective blocking of CP-AMPARs may cause disinhibition and further shift the excitation–inhibition balance toward excitation. On the other hand, CP-AMPARs are transiently upregulated in many epilepsy models (Rajasekaran et al., 2012; Joshi et al., 2017; Amakhin et al., 2018), and their block in this context may be beneficial. Different AMPA receptor antagonists differentially affect two main classes of AMPA receptors: calcium-permeable and calcium-impermeable. For instance, many polyamine toxins and dicationic adamantane and phenylcyclohexyl derivatives (Magazanik et al., 1997; Mellor and Usherwood, 2004; Bolshakov et al., 2005) are more active against calcium-permeable class, while pentobarbital is more selective against calcium-impermeable AMPA receptors (Taverna et al., 1994; Yamakura et al., 1995). In contrast, perampanel equipotently inhibits CP- and CI-AMPARs (Barygin, 2016; Fukushima et al., 2020). Thus, we decided to compare the action of antiseizure drugs on calcium-permeable and calcium-impermeable AMPA receptors.

In our experiments, lamotrigine, topiramate, levetiracetam, felbamate, carbamazepine, tiagabin, vigabatrin, zonisamide, and gabapentin did not demonstrate strong activity against CP- and CI-AMPARs indicating that these receptors do not play a significant role in their pharmacological profile. In contrast, phenytoin inhibited both major AMPA receptor subtypes, being much more active against CI-AMPARs (IC50 = 30 ± 4 µM) than against CP-AMPARs (250 ± 60 µM). The main target for phenytoin in the brain, which is believed to underlie its anticonvulsant properties, are voltage-gated sodium channels. Affinity of phenytoin to inactivated states of sodium channels is in the range of 7–21 µM (Kuo and Bean, 1994; Lenkowski et al., 2007). Thus, affinity of phenytoin to CI-AMPARs is only slightly lower than affinity to its primary target. Analysis of molecular mechanisms of action of phenytoin on AMPARs demonstrated close similarity with those of pentobarbital. The hypothesis about a common site is further supported by 3D similarity between these two compounds. Our data suggest that inhibition of CI-AMPARs is essential for phenytoin anticonvulsant effects.

## Materials and Methods

All experimental procedures were approved by the Animal Care and Use Committee of the I.M. Sechenov Institute of Evolutionary Physiology and Biochemistry of the Russian Academy of Sciences. Wistar rats (13–19 days old) were anesthetized with sevoflurane and then decapitated. Maximum effort was made to minimize the number of animals used. Brains were removed quickly and cooled to 2–4°C in an ice bath. Transverse hippocampal and striatal slices (250 µm thick) were prepared using a vibratome (Campden Instruments Ltd, Loughborough, United Kingdom) and stored in a solution containing (in mM) 124 NaCl, 5 KCl, 1.3 CaCl_2_, 2.0 MgCl_2_, 26 NaHCO_3_, 1.24 NaH_2_PO_4_, and 10 D-glucose, aerated with carbogen (95% O_2_, 5% CO_2_). Single neurons were freed from slices by vibrodissociation (Vorobjev, 1991). The antagonism of CP-AMPARs was studied on striatal giant interneurons (Bernard et al., 1997; Gotz et al., 1997), which were identified by their shape and size. They have large (>25 µm) soma of polygonal shape, whereas principal cells are significantly smaller and nearly spherical. Previous works demonstrated that a nondesensitizing response to kainate in these neurons is mediated by GluA2-lacking AMPARs. The sensitivity to dicationic blockers like IEM-1460, IEM-1925, and polycationic toxins agrees with the data on recombinant receptors (Bolshakov et al., 2005; Barygin et al., 2011) The currents demonstrate inward rectification and significant Ca^2+^ permeability (Buldakova et al., 1999; Samoilova et al., 1999). The antagonism of CI-AMPARs was studied on pyramidal neurons from the CA1 area of the hippocampus. Kainate-induced currents in these neurons are virtually insensitive to cationic blockers (Magazanik et al., 1997; Bolshakov et al., 2005).

The whole-cell patch-clamp technique was used for recording of membrane currents generated in response to applications of kainate. Series resistance of about 20 MΩ was compensated by 70–80% and monitored during experiments. Currents were recorded using an EPC-8 amplifier (HEKA Electronics, Lambrecht, Germany), filtered at 5 kHz, sampled, and stored on a personal computer. Drugs were applied using the RSC-200 perfusion system (BioLogic Science Instruments, Claix, France) under computer control. The solution exchange time in the whole-cell mode was 50–60 ms. The extracellular solution contained (in mM) NaCl 143, KCl 5, MgCl_2_ 2.0, CaCl_2_ 2.5, D-glucose 18, and HEPES 10 (pH was adjusted to 7.3 with HCl). The pipette solution contained (in mM) CsF 100, CsCl 40, NaCl 5, CaCl_2_ 0.5, EGTA 5, and HEPES 10 (pH was adjusted to 7.2 with CsOH). Experiments were performed at room temperature (22–24°C). Phenytoin sodium (PHR1492) was purchased from Sigma (St Louis, MO, United States), as well as hydantoin, 5-benzylhydantoin, primidone, and ethosuximide. Lamotrigine, topiramate, levetiracetam, felbamate, carbamazepine, tiagabin, vigabatrin, zonisamide, and gabapentin were purchased from Tocris Bioscience (Bristol, United Kingdom). Perampanel was from MedChemExpress (Stockholm, Sweden). Kinetics of transient processes of more than 20-ms duration was approximated by exponential functions. Experiments were performed at −80 mV holding voltage. All experimental data are presented as mean ± SD estimated from at least four experiments. Significance of the effects was tested with paired *t* test. Differences were considered significant at *p* < 0.05. 3D structures of compounds were calculated by the ZMM software (zmmsoft.ca).

## Results

### Screening for Activity Against AMPA Receptors Among Anticonvulsants

Application of 100 µM kainate on isolated hippocampal CA1 pyramidal neurons (CI-AMPARs) and giant striatal interneurons (CP-AMPARs) induced weakly or nondesensitizing inward currents. We initially checked if these kainate-induced currents will be inhibited by different anticonvulsants at 100-µM concentrations. In our experiments, lamotrigine, topiramate, levetiracetam, felbamate, carbamazepine, tiagabin, vigabatrin, zonisamide, and gabapentin were practically inactive (inhibition ≤20%, Table 1) against both CP-AMPARs and CI-AMPARs. These data agree well with the results of Fukushima et al. (2020), who also did not find significant activity of different anticonvulsants (topiramate, phenobarbital, lamotrigine, gabapentin, carbamazepine, valproate, levetiracetam, and lacosamide), except perampanel, against hGluA1-4 receptors. In contrast, phenytoin reversibly inhibited both CP-AMPARs and CI-AMPARs in our experiments, and we decided to study its molecular mechanisms of action in more detail. Fukushima et al. (2020) did not test phenytoin in their paper.

**TABLE 1:** Action of anticonvulsants on CP- and CI-AMPARs

Anticonvulsant	CP-AMPARs% inhibition at 100 µM	CI-AMPARs% inhibition at 100 µM
Lamotrigine	13 ± 3	14 ± 5
Topiramate	14 ± 2	12 ± 2
Levetiracetam	9 ± 3	6 ± 2
Felbamate	10 ± 4	16 ± 4
Carbamazepine	15 ± 4	11 ± 3
Tiagabin	10 ± 2	14 ± 2
Vigabatrin	10 ± 3	8 ± 1
Zonisamide	11 ± 3	11 ± 2
Gabapentin	12 ± 5	20 ± 6

N = 5 for each compound both against CP-AMPARs and CI-AMPARs. The effect of all compounds on CP-AMPARs and CI-AMPARs was significant (*p* < 0.05, paired t test).

### Concentration Dependence of Action of Phenytoin on Calcium-Impermeable and Calcium-Permeable AMPARs

**FIGURE 1:**
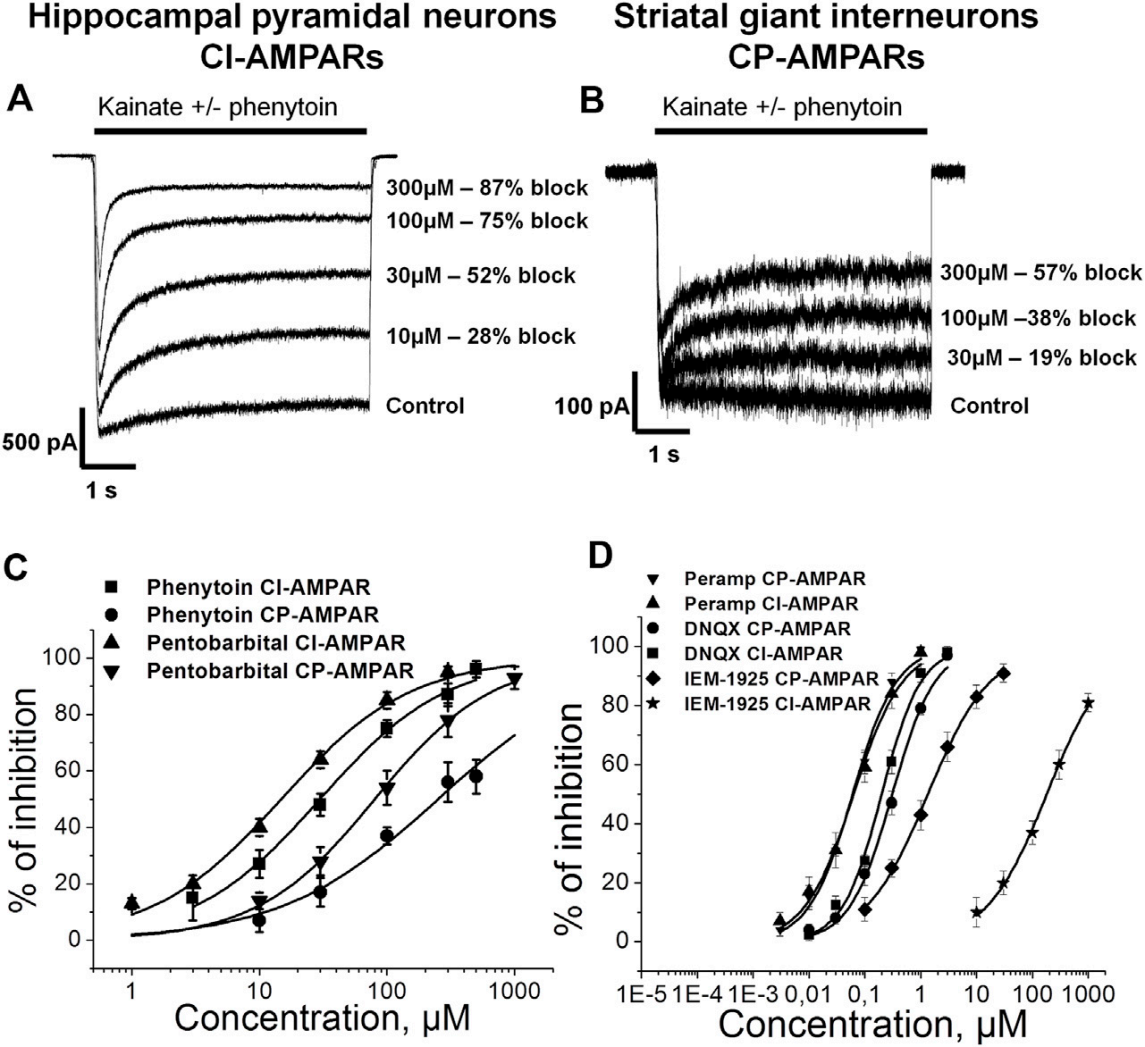
Concentration dependence of action of phenytoin on calcium-impermeable and calcium-permeable AMPARs. **(A,B)** Representative examples of CI-AMPARs **(A)** and CP-AMPARs **(B)** inhibition by different concentrations of phenytoin. **(C,D)** Concentration-inhibition curves for phenytoin, pentobarbital **(C)**, and major AMPARs antagonists **(D)**.

Representative recordings demonstrating the action of different phenytoin concentrations on CI-AMPARs of hippocampal pyramidal neurons and CP-AMPARs of striatal giant interneurons are shown (Figures 1A,B). At the highest concentration tested—500 µM—phenytoin demonstrated almost complete inhibition of kainate-induced currents in hippocampal pyramidal neurons and only 58 ± 5% inhibition in striatal giant interneurons. Because of the poor solubility of phenytoin in the extracellular solution, we were not able to test higher concentrations. The IC50 value for CI-AMPARs obtained using the Hill equation was 30 ± 4 µM, and the Hill coefficient was 0.9 ± 0.2. For CP-AMPARs, approximation by the Hill equation gave IC50 value = 250 ± 60 µM and Hill coefficient = 0.7 ± 0.2 (Figure 1C). Thus, we have shown for the first time that phenytoin is more active against CI-AMPARs. Among known AMPAR antagonists, similar preference for CI-AMPARs demonstrated pentobarbital (Taverna et al., 1994; Yamakura et al., 1995; Jackson et al., 2003). So we decided to compare its activity in experiments on hippocampal CA1 pyramidal neurons and giant striatal interneurons (Figure 1C). Indeed, in our experiments, the IC50 values for pentobarbital were 14 ± 3 and 80 ± 13 µM for CI-AMPARs and CP-AMPARs, respectively. Figure 1D illustrates concentration dependencies of action of representatives of three major types of AMPARs antagonists—competitive antagonist DNQX (Honore et al., 1988), negative allosteric antagonist perampanel (Hanada et al., 2011; Chen et al., 2014), and use and voltage-dependent channel blocker IEM-1925—on CI- and CP-AMPARs. DNQX was slightly more active against hippocampal CI-AMPARs, perampanel equipotently inhibited both receptor subtypes (Barygin, 2016), and IEM-1925 was dramatically more active against CP-AMPARs. The IC50 values are provided in Table 2.

**TABLE 2:** Characteristic features of AMPAR inhibition by different antagonists

Compound/Feature	Phenytoin	DNQX	Perampanel	IEM-1925	Pentobarbital
More active against CI-AMPARs	Yes	Yes	No	No	Yes
IC50 CI-AMPARs	30 ± 4 M	0.20 ± 0.03 µM	63 ± 8 nM	180 ± 30 µM	14 ± 3 µM
IC50 CP-AMPARs	250 ± 60 µM	0.31 ± 0.06 µM	60 ± 6 nM	1.3 ± 0.4 µM	80 ± 13 µM
Activity drop at high (500 µM) kainate concentration	Yes	Yes	Yes	No	Yes
Activity drop in the presence of cyclothiazide	Yes	Yes	Yes	N.D.	Yes
Trap in kainate 100 µM	Yes	N.D.	No	Yes	Yes
Trap in kainate 500 µM	?	N.D.	No	Yes	N.D.
Competition with phenytoin for binding site	Not applicable	No	No	N.D.	N.D.
Difference in the % of inhibition in coapplication and preapplication protocols	Yes	N.D.	No	Yes	Yes
pH-dependence	Yes	Yes^a^	N.D.	N.D.	Yes

a Dudic and Reiner, 2019.

### Action of Compounds Structurally Related to Phenytoin on CI-AMPARs

**FIGURE 2:**
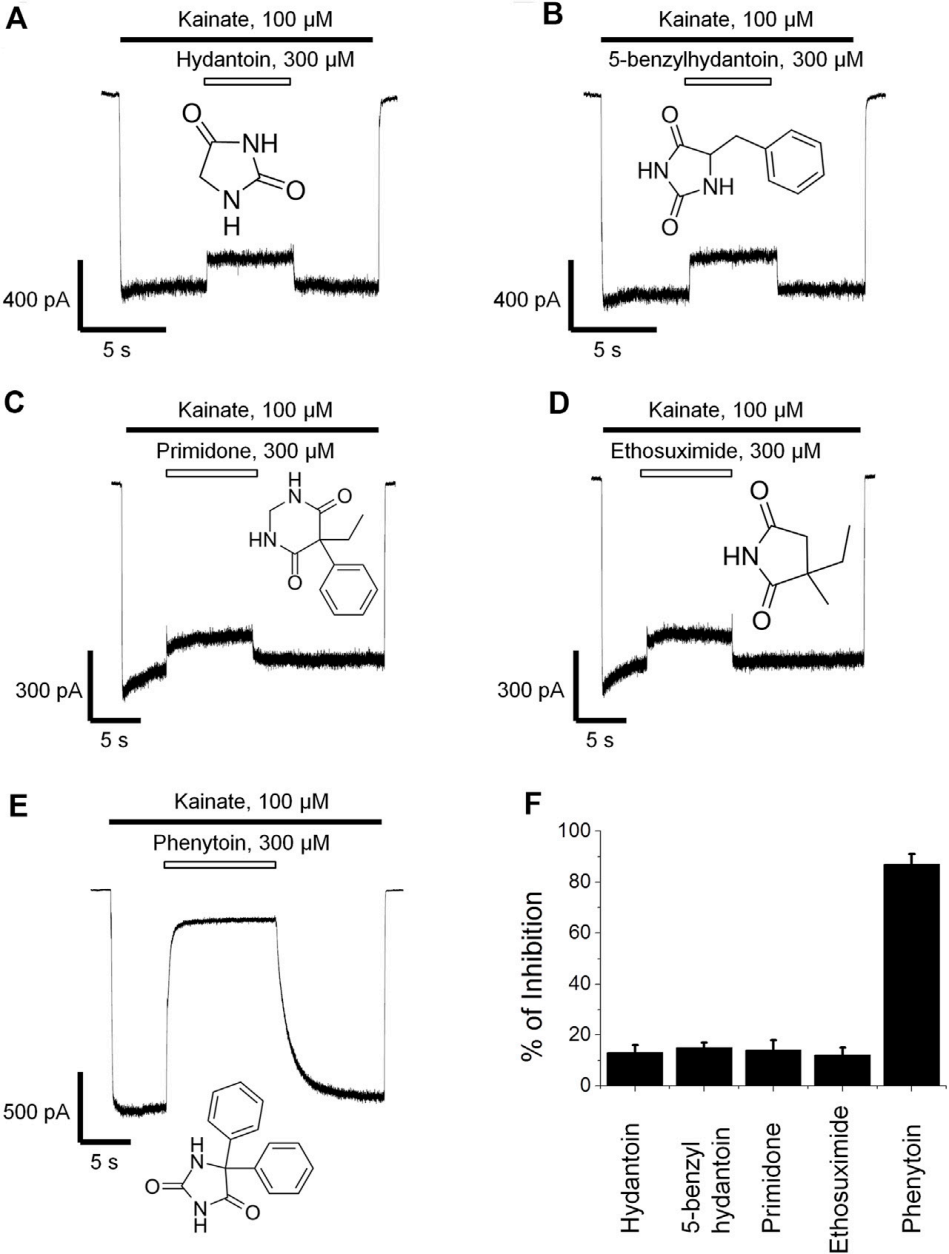
Action of compounds structurally related to phenytoin on CI-AMPARs. Representative examples of weak inhibition by 300 µM hydantoin **(A)**, 5-benzylhydantoin **(B)**, primidone **(C)**, and ethosuximide **(D)** and strong inhibition by 300 µM phenytoin **(E)**. **(F)** Summarized results of AMPARs inhibition by these compounds at 300-µM concentrations.

Phenytoin is a diphenyl derivative of hydantoin. So we decided to test whether hydantoin itself or 5-benzylhydantoin will be able to inhibit CI-AMPARs. Both compounds demonstrated only weak activity even at high 300-µM concentration (Figures 2A,B). Primidone and ethosuximide—two anticonvulsant compounds structurally related to phenytoin—were only weakly active as well (Figures 2C,D). The percentage of inhibition by hydantoin, 5-benzylhydantoin, primidone, and ethosuximide at 300-µM concentration did not exceed 20%. A representative example of strong (≥80%) inhibition by 300 µM phenytoin is provided for comparison (Figure 2E). The inhibitory action of compounds is summarized in the bar graph in Figure 2F.

### The Action of Phenytoin Is Attenuated in Experiments With High Kainate Concentrations but Is Not Competitive

**FIGURE 3:**
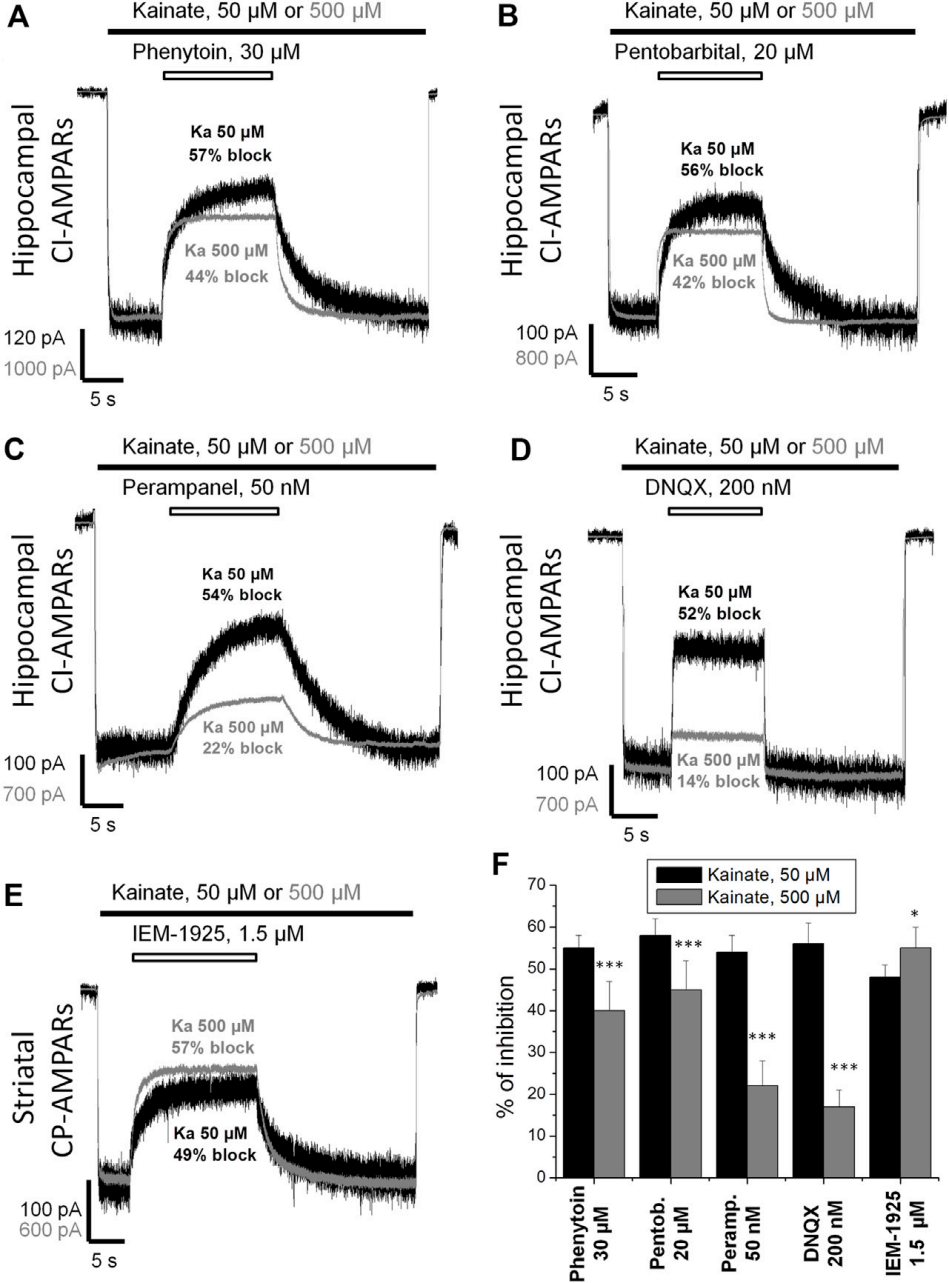
Kainate concentration dependence of action of compounds on AMPA receptors. **(A–E)** Representative examples of CI-AMPARs inhibition by 30 µM phenytoin **(A)**, 20 µM pentobarbital **(B)**, 50 nM perampanel **(C)**, 200 nM DNQX **(D)**, and CP-AMPARs inhibition by 1.5 µM IEM-1925 **(E)** at 50 and 500 µM kainate concentrations. The amplitudes of responses at different kainate concentrations were normalized for visual clarity. **(F)** Summarized results of AMPARs inhibition by different compounds at 50 and 500 µM kainate concentrations. Phenytoin, pentobarbital, perampanel, and DNQX were more active in case of lower kainate concentration. In contrast, IEM-1925 was more active in case of higher kainate concentration. *–*p* < 0.05. ***–*p* < 0.001.

Kawano et al. (1994) suggested that the action of phenytoin on AMPA receptors is competitive. So we initially compared the percentage of inhibition by phenytoin at two different kainate concentrations—50 and 500 µM (Figure 3). Indeed, in hippocampal pyramidal neurons, 30 µM phenytoin stronger inhibited currents induced by 50 µM kainate concentration, demonstrating 55 ± 3% inhibition, against 40 ± 7% at 500 µM kainate concentration (*n* = 7, *p* < 0.001). Likewise, 200 µM phenytoin stronger inhibited currents in striatal giant interneurons induced by 50 µM kainate concentration, demonstrating 59 ± 5% inhibition, against 48 ± 2% at 500 µM kainate concentration (*n* = 5, *p* < 0.01, data not shown). In this and further series of experiments, we used pentobarbital, DNQX, perampanel, and IEM-1925 as reference agents. The decrease in inhibitory activity with the increase in kainate concentration in the range from 50 to 3,000 µM was demonstrated for pentobarbital earlier (Jackson et al., 2003). In our experiments on hippocampal CI-AMPARs, 20 µM pentobarbital was also more active in the case of lower kainate concentration (Figure 3B), as well as 50 nM perampanel (Figure 3C) and 200 nM DNQX (Figure 3D). In contrast, IEM-1925 (Figure 3E) stronger inhibited currents induced by 500 µM kainate (55 ± 5%), then by 50 µM kainate (48 ± 3%) on striatal CP-AMPARs. The bar graph in Figure 3F summarizes the obtained results. Phenytoin and pentobarbital demonstrated moderate (10–15%) decrease in the % of inhibition with the increase in agonist concentration, while for perampanel and DNQX, the decrease was stronger (30–40%).

**FIGURE 4:**
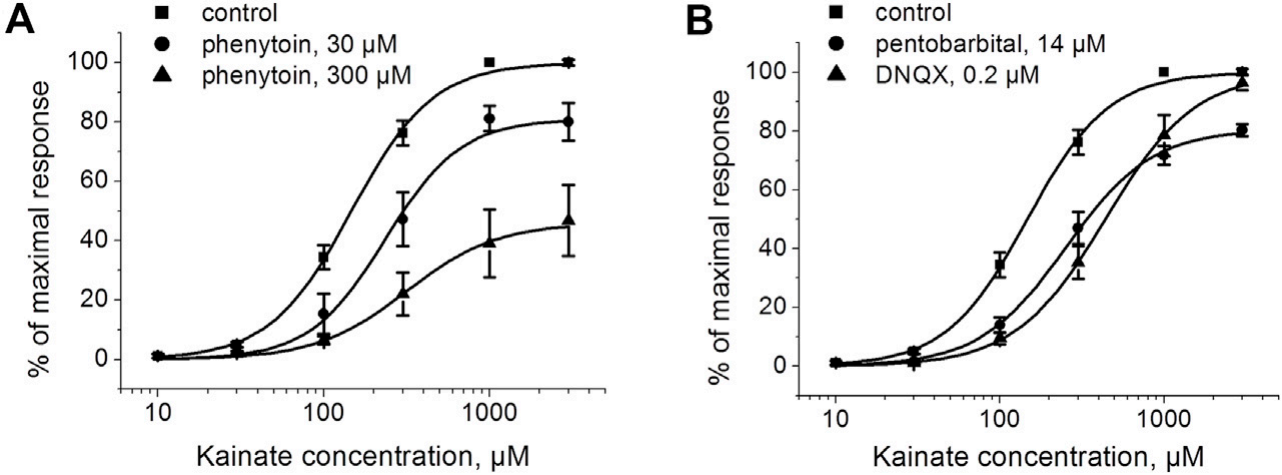
The action of phenytoin is not competitive. **(A)** Activation curve for kainate in control and in the presence of 30 and 300 μM phenytoin. Phenytoin in both concentrations reduced the maximal response to kainate, which suggests that inhibition by phenytoin is not competitive. **(B)** Activation curve for kainate in the absence and presence of 14 μM pentobarbital and 0.2 μM DNQX. Pentobarbital demonstrated inhibition even at high kainate concentrations as well, while DNQX induced a parallel shift of the kainate activation curve, which is typical for competitive inhibitors.

To further test whether inhibition by phenytoin is competitive or not, we studied the kainate concentration dependence in the absence and in the presence of phenytoin, 30 and 300 µM (Figure 4A). The EC50 for kainate was 150 ± 20 µM in control, and the Hill coefficient was 1.6 ± 0.2. The EC50 value was increased to 250 ± 30 μM and 360 ± 30 µM in the presence of 30 and 300 µM phenytoin, respectively. Maximal response to kainate was reduced to 84 ± 4% by 30 µM phenytoin and to 46 ± 3% by 300 µM phenytoin (*n* = 5 for both phenytoin concentrations, *p* < 0.001), clearly indicating that inhibition by phenytoin is not competitive. Pentobarbital of 14 µM decreased the maximal response to kainate as well (Figure 4B), while 0.2 µM DNQX did not change it (Figure 4B), which is typical for competitive inhibitors.

### The Effect of Phenytoin Is Attenuated in the Presence of Cyclothiazide

**FIGURE 5:**
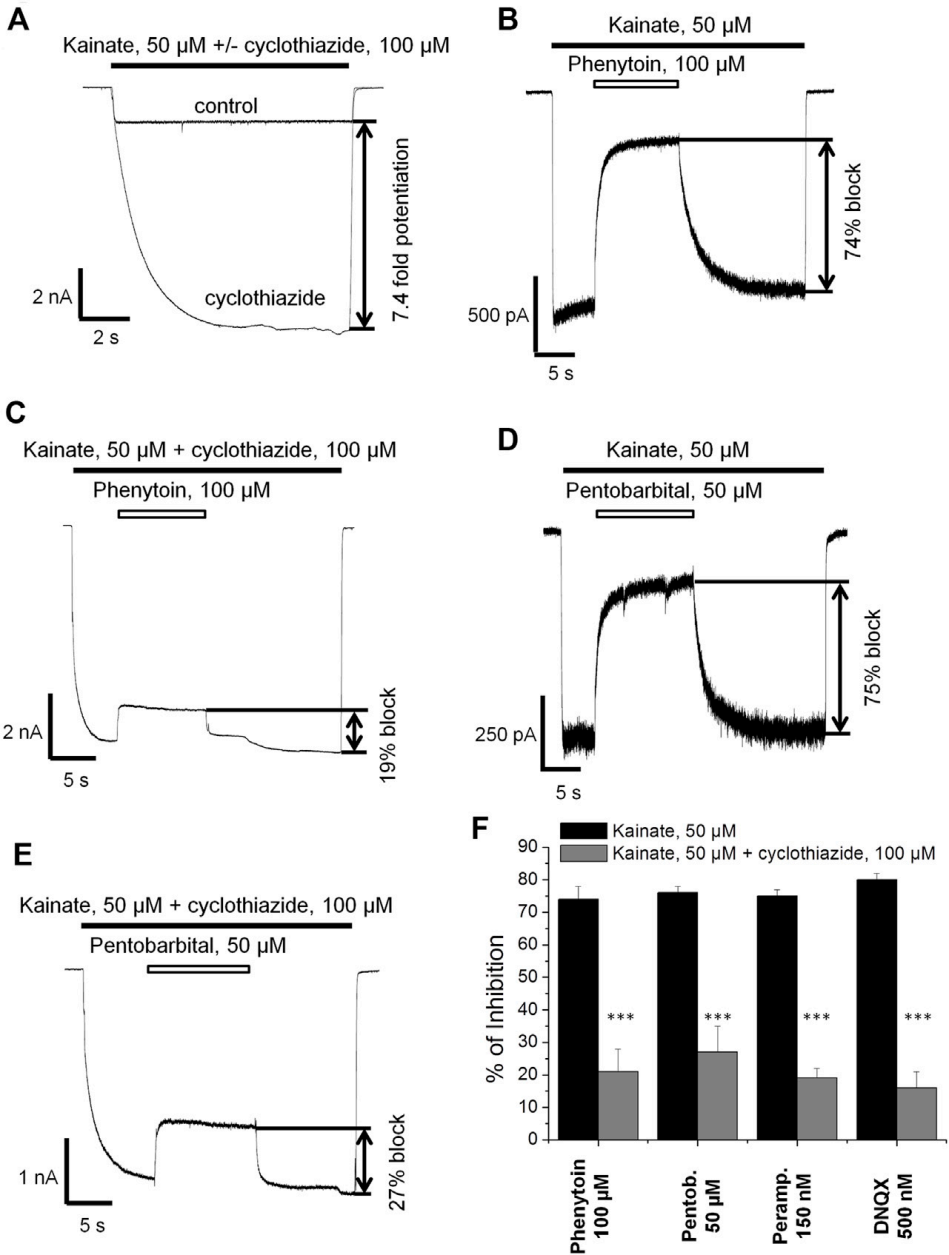
The effect of phenytoin is attenuated in the presence of cyclothiazide. **(A)** Enhancement of kainate-induced currents in hippocampal CA1 pyramidal neurons by 100 µM cyclothiazide. **(B,C)** Representative examples of inhibition by 100 µM phenytoin in the absence **(B)** and presence **(C)** of 100 µM cyclothiazide. **(D,E)** Representative examples of inhibition by 50 µM pentobarbital in the absence **(D)** and presence **(E)** of 100 µM cyclothiazide. **(F)** Summarized results of CI-AMPARs inhibition by different compounds in the absence and presence of 100 µM cyclothiazide. The inhibitory effect of compounds was significantly attenuated in the presence of cyclothiazide. ***–*p* < 0.001.

Next we decided to compare the action of phenytoin in the presence and absence of cyclothiazide, a positive AMPAR allosteric modulator. Cyclothiazide is mostly known as an agent that reduces AMPA receptor desensitization (Partin et al., 1993; Patneau et al., 1993). It also demonstrates slow onset increase in the steady-state current amplitudes and lengthens single-channel openings (Patneau et al., 1993; Fucile et al., 2006). Cyclothiazide strongly increases AMPAR currents in hippocampal CA1 pyramidal cells but only weakly affects those of giant striatal interneurons (Buldakova et al., 2000). Thus, we decided to study the effect of phenytoin (100 µM) at relatively low kainate concentration (50 µM) in the absence or presence of saturating concentration of cyclothiazide (100 µM) on hippocampal CA1 pyramidal neurons. Cyclothiazide of 100 µM increased the stationary current induced by 50 µM kainate by 8 ± 2 fold (Figure 5A). Representative examples of inhibition by 100 µM phenytoin in the absence and presence of 100 µM cyclothiazide are shown (Figures 5B,C). Phenytoin of 100 µM was drastically more active in the absence than in the presence of cyclothiazide (74 ± 4% vs. 21 ± 7% inhibition, respectively; *p* <0.001). The inhibitory effect of 50 µM pentobarbital (Figure 5D) was also significantly attenuated in the presence of cyclothiazide (Figure 5E) in line with previous results (Jackson et al., 2003). DNQX was less active in the presence of cyclothiazide (data not shown), as well as perampanel (Barygin, 2016). Because cyclothiazide has only weak effect on CP-AMPARs of giant striatal interneurons, we decided not to test IEM-1925 in this protocol. The bar graph in Figure 5F summarizes the obtained results.

### Trapping of Phenytoin in AMPAR Channels

Up to this point, the mechanisms of action of phenytoin closely resembled that of pentobarbital (preference for CI-AMPAR, the decrease in inhibitory activity in experiments with high kainate concentrations and in the presence of cyclothiazide). A distinctive feature of the open channel blockers of AMPARs, like IEM-1925, is the trapping effect. The blocked channels can close after agonist dissociation trapping the blocker molecules inside (Bahring and Mayer, 1998; Tikhonova et al., 2008). Pentobarbital demonstrated trapping in closed AMPA receptor channels that was stable over time but was much weaker in the presence of cyclothiazide (Jackson et al., 2003).

**FIGURE 6:**
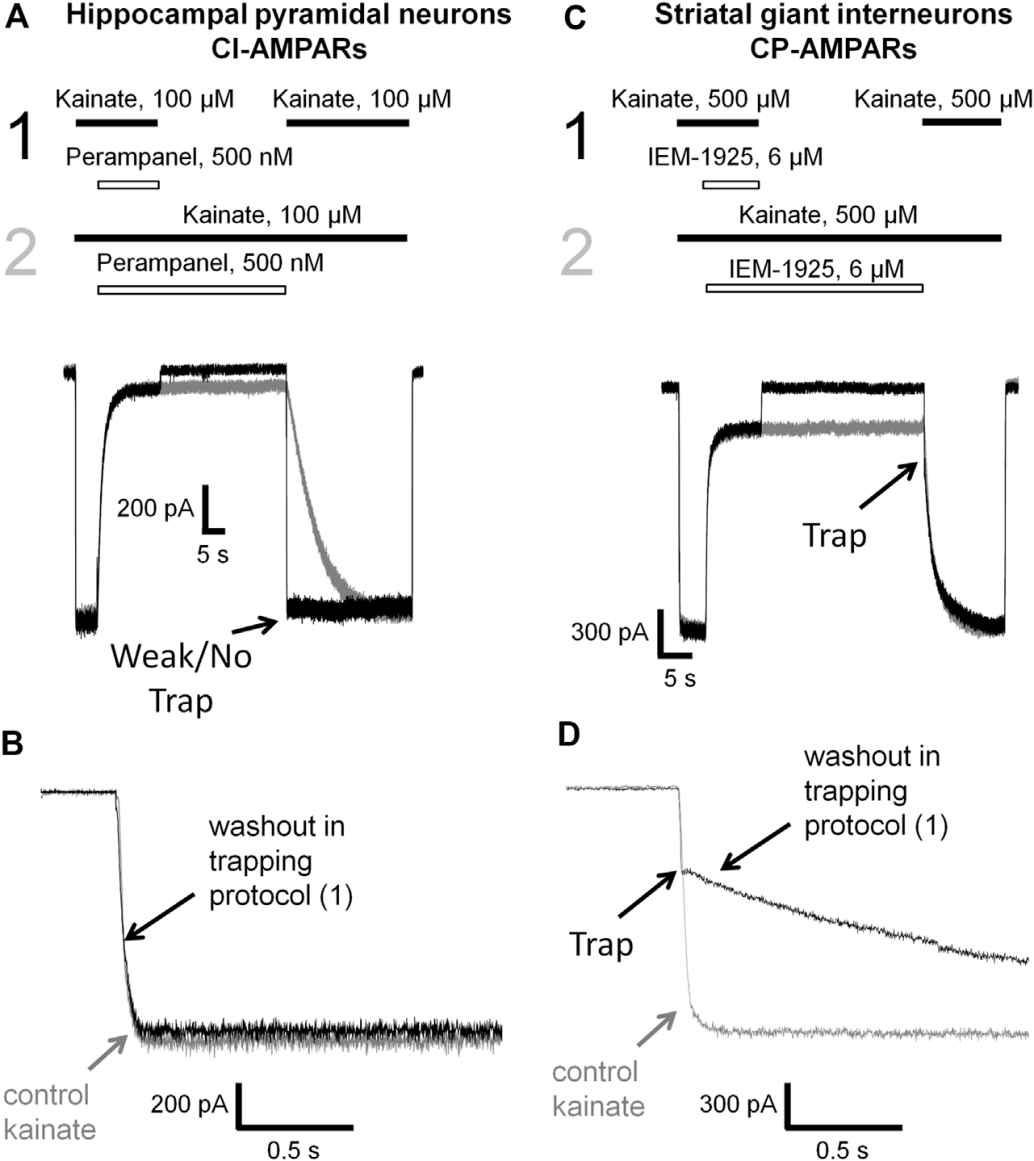
Perampanel is not trapped in closed AMPAR channels in contrast to IEM-1925. **(A)** Representative example of the absence of trapping for 500 nM perampanel in the double-pulse protocol (black traces) and its recovery kinetics in the protocol without pause in extracellular solution (gray traces) on hippocampal pyramidal neuron. **(B)** Superimposition of rising fronts in control kainate application and testing kainate application in the double-pulse protocol for 500 nM perampanel. **(C)** Representative example of trapping for 6 µM IEM-1925 in the double-pulse protocol (black traces) and its recovery kinetics in the protocol without pause in extracellular solution (gray traces) on striatal giant interneuron. **(D)** Superimposition of rising fronts in control kainate application and testing kainate application in the double-pulse protocol for 6 µM IEM-1925.

**FIGURE 7:**
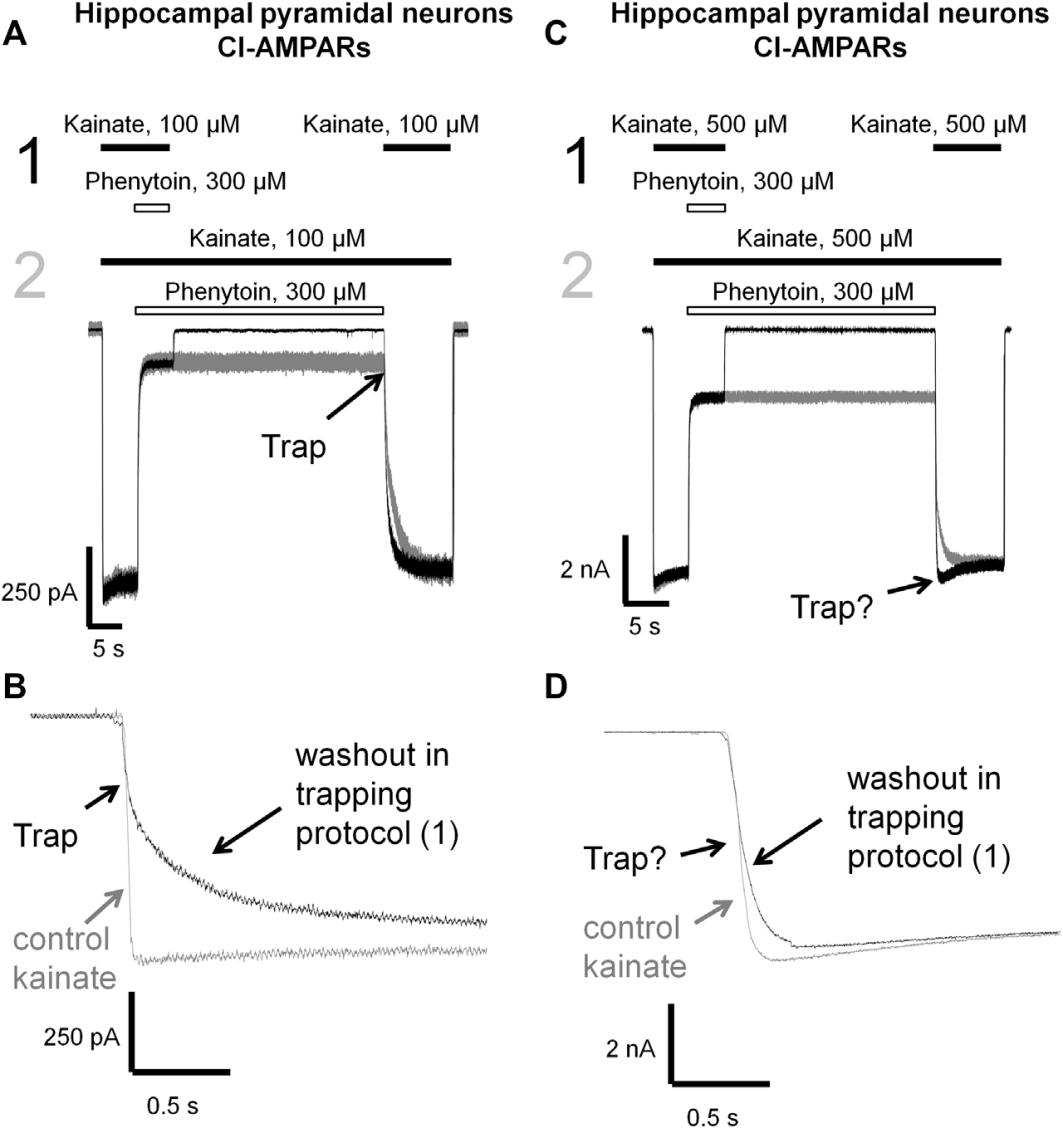
Trapping of phenytoin in AMPAR channels. **(A,C)** Representative examples of phenytoin trapping in case of CI-AMPAR activation by 100 **(A)** and 500 µM **(C)** kainate in the double-pulse protocol (black traces) and recovery kinetics in the protocol without pause in extracellular solution (gray traces). Phenytoin of 300 µM demonstrated pronounced trapping at 100 µM kainate **(A)**, but trapping was questionable at 500 µM kainate **(C)**. **(B,D)** Superimpositions of rising fronts in control kainate application and testing kainate application in the double-pulse protocol in case of CI-AMPAR activation by 100 **(B)** and 500 µM **(D)** kainate.

Here we decided to compare phenytoin trapping in case of CI-AMPAR activation by 100 and 500 µM kainate using the double-pulse protocol (Huettner and Bean, 1988; Blanpied et al., 1997). In this protocol, denoted as protocol 1 (black traces) in Figures 6, 7, we initially apply kainate, then add an antagonist, then simultaneously remove both kainate and antagonist for a 30-s pause in the extracellular solution, and finally apply the testing kainate to study the recovery kinetics. If the response to testing kainate application resembles that of the first kainate application, then we can say that the antagonist was not trapped in the closed channels. If it includes a slower component, we can say that some molecules of the antagonist were trapped. Recovery kinetics for the protocol in which the 30-s pause in the extracellular solution is changed to 30 s in the presence of both kainate and antagonist (protocol 2, gray traces) is provided for comparison.

Kinetics of control kainate response is single-exponential (*τ* = 20–50 ms in different conditions). For perampanel (Figures 6A,B), the kinetics of the response to testing kainate application in the trapping protocol was also single-exponential, and the time constant (*τ* = 41 ± 12 ms, *n* = 4) was not significantly different from that of the control kainate response (*τ* = 43 ± 9, *n* = 4, *p* ≥ 0.05), evidencing the absence of trapping effect. In contrast, IEM-1925 demonstrated at least double-exponential kinetics: the fast component was close to that of the kainate control, while the slow one (1,800 ± 400 ms, *n* = 4) did not differ significantly from recovery kinetics in protocol 2 (1,900 ± 500 ms, *n* = 4, *p* ≥0.05), indicating trapping (Figures 6C,D).

Phenytoin in 100 µM kainate behaved similar to IEM-1925, demonstrating at least double-exponential washout kinetics in the trapping protocol with the fast component coinciding with the kinetics of the control response (Figures 7A,B). Thus, phenytoin demonstrated pronounced trapping in case of AMPAR activation with 100 µM kainate. The situation in 500 µM kainate was markedly different (see Figures 7C,D). The kinetics of the testing response to kainate was well fitted by a single exponential function (*τ* = 81 ± 17 ms, *n* = 4), which was significantly slower than the kinetics of control kainate (*τ* = 39 ± 13 ms *n* = 4, *p* < 0.05). However, it was fivefold faster than recovery from phenytoin block in protocol 2 (*τ* = 390 ± 60 ms, *n* = 4, *p* <0.01). Unambiguous conclusion is not possible in this situation, but the obvious difference between Figures 7B,D suggests that phenytoin trapping is dependent on kainate concentration.

### Absence of Competition of Phenytoin With Competitive Antagonists and Negative Allosteric Antagonists

**FIGURE 8:**
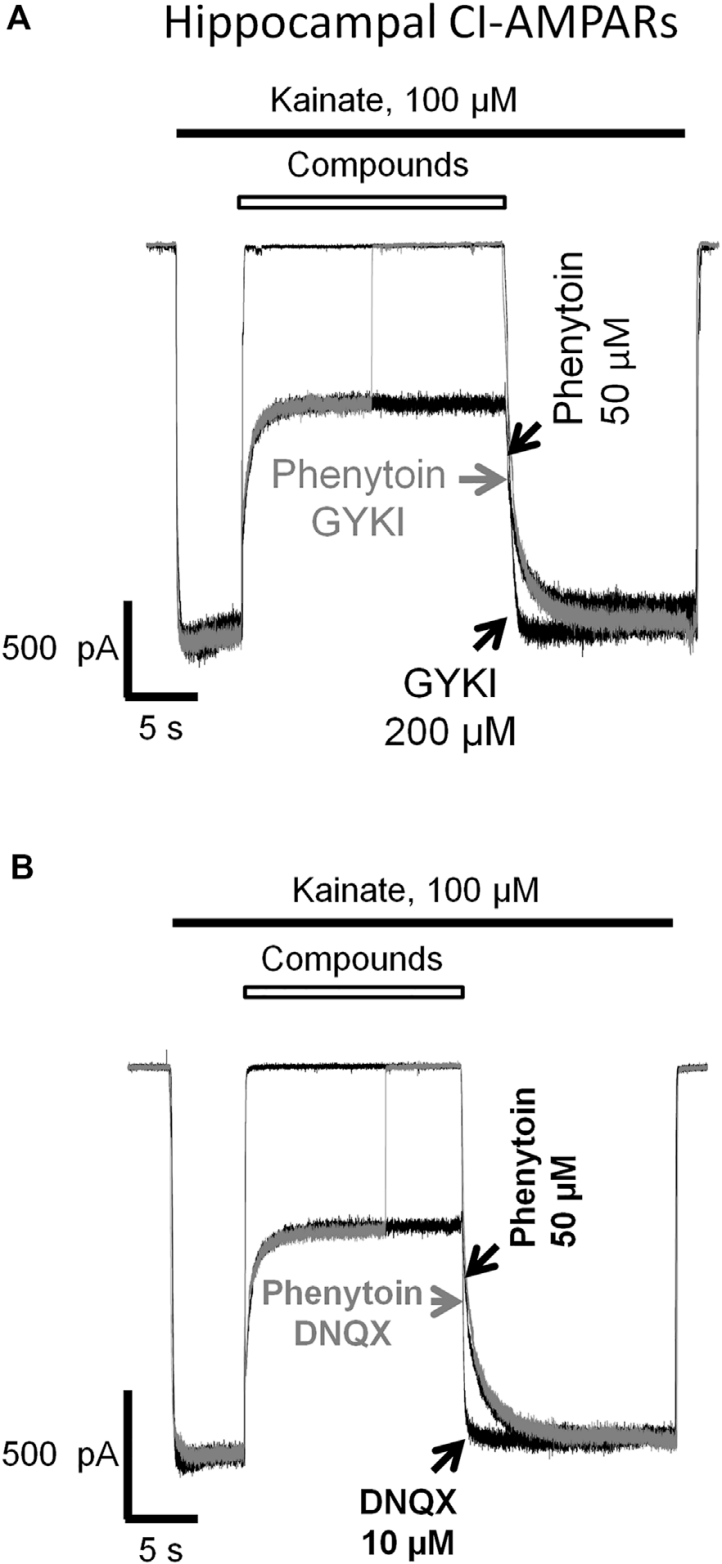
Absence of competition of phenytoin with competitive and negative allosteric AMPA receptor antagonists. The slow phase of recovery from 50 µM phenytoin (black trace) remains unchanged in the presence of 200 µM negative allosteric antagonist GYKI-52466 **(A)** and 10 µM competitive antagonist DNQX **(B)** (gray trace) suggesting that the site of action of phenytoin is different from that of competitive and negative allosteric AMPAR antagonists.

In our experiments (Figures 1, 3, 4, 5, 6, 7), phenytoin demonstrated features that discriminated it from classical types of AMPAR antagonists (competitive antagonists, negative allosteric antagonists, CP-AMPARs selective channel blockers). However, it was somewhat similar to that of competitive and negative allosteric antagonists because all these compounds were less active in conditions, resulting in strong AMPAR activation (high agonist concentration or presence of cyclothiazide). To further ensure that this is the case, we performed direct experiments on competition for the same site of action with phenytoin and abovementioned types of ligands using the difference in recovery kinetics. Washout kinetics for 50 µM phenytoin is relatively slow, τ = 1,100 ± 200 ms for CI-AMPARs of hippocampal pyramidal neurons. To study the competition, we used excessive concentrations of “fast” negative allosteric antagonist GYKI-52466 or “fast” competitive antagonist DNQX. Experiments on the competition of phenytoin with GYKI-52466 (Figure 8A) and DNQX (Figure 8B) were performed on hippocampal pyramidal neurons. We initially studied the washout kinetics of each compound alone and then compared it with washout kinetics in the complex protocol, where we initially applied 50 µM phenytoin, and then added a mixture of phenytoin and excessive concentration of a “fast” antagonist. Indeed, if any fast antagonist would be able to displace phenytoin or somehow affect its binding, the washout kinetics in the complex protocol would be faster than that for phenytoin alone. Neither 200 µM GYKI-52466 nor 10 µM DNQX affected the kinetics of phenytoin washout in this complex protocol. On the other hand, fast negative allosteric antagonist GYKI-52466 was able to displace slow negative allosteric antagonist perampanel in our earlier experiments (Barygin, 2016). These data suggest that the binding site of phenytoin in CI-AMPARs is different from that of competitive antagonists and negative allosteric antagonists. Further studies using site-directed mutagenesis and cryo-electron microscopy/X-ray crystallography are needed to map it.

### Phenytoin Preferentially Binds to the Open Channels and Is More Active at pH 7.4 Comparing to pH 9.0

**FIGURE 9:**
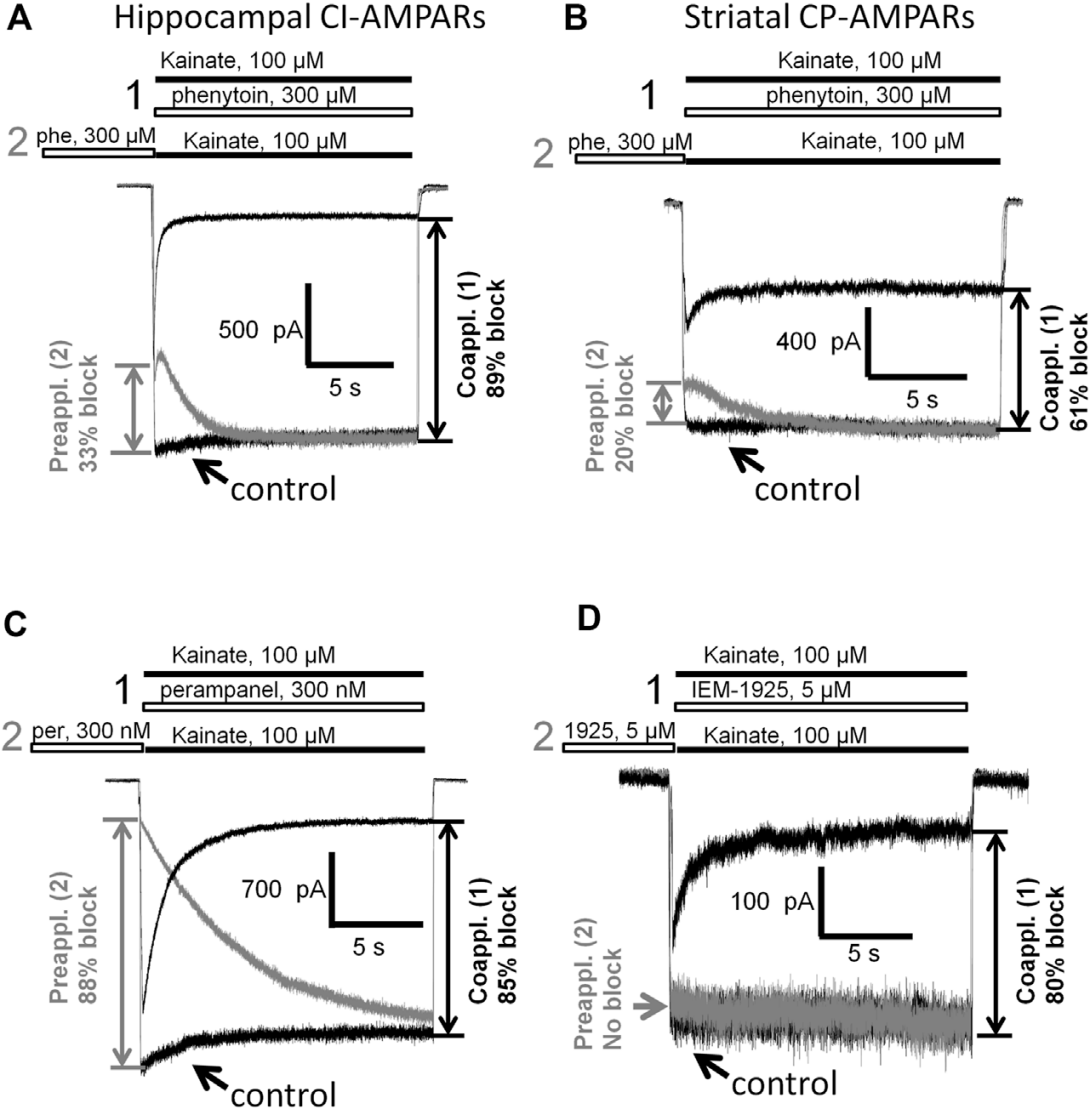
Closed and open channel AMPAR inhibition by phenytoin, perampanel, and IEM-1925. **(A,B)** Comparison of the effects of 300 µM phenytoin on CI-AMPARs of hippocampal pyramidal neurons **(A)** and CP-AMPARs of striatal giant interneurons **(B)** in case of coapplication with kainate (black traces) and preapplication without kainate (gray traces). For both receptor types, phenytoin is more effective in the coapplication protocol. **(C)** Comparison of the effects of 300 nM perampanel on CI-AMPARs of hippocampal pyramidal neurons in case of coapplication with kainate (black trace) and preapplication without kainate (gray trace). Perampanel is equally effective in these two protocols. **(D)** Comparison of the effects of 5 µM IEM-1925 on CP-AMPARs of striatal giant interneurons in case of coapplication with kainate (black trace) and preapplication without kainate (gray trace). IEM-1925 is effective only in the coapplication protocol.

Having shown that the mechanisms of action of phenytoin on AMPA receptors do not resemble those of competitive antagonists, negative allosteric antagonists, and CP-AMPARs selective channel blockers, we decided to further investigate them. So we compared the action of phenytoin on open (protocol 1, coapplication with agonist) and closed (protocol 2, preapplication without agonist) AMPAR channels (Figure 9). In experiments with hippocampal CI-AMPARs, 300 µM phenytoin was able to inhibit both closed (37 ± 7%) and open AMPA receptor channels (87 ± 5%), demonstrating preference for open channels (Figure 9A, *n* = 5, *p* < 0.001). Similar preference for open channels was found in experiments with CP-AMPARs of giant striatal interneurons. Phenytoin of 300 µM blocked 60 ± 6% in case of coapplication with agonist and 24 + 9% in case of preapplication without agonist (Figure 9B, *n* = 8, *p* < 0.001). Pentobarbital of 100 µM was also more active in the coapplication protocol in the experiment on hippocampal CI-AMPARs (data not shown). In contrast, 300 nM perampanel was equally effective in preapplication and coapplication protocols on hippocampal CI-AMPARs (Figure 9C). At a glance, this result contradicts with previous data, suggesting that perampanel binds to the resting receptors more efficiently than to activated ones (Yelshanskaya et al., 2016). However, this conclusion was made from experiments with recombinant GluA2 AMPA receptors that were done in the presence of cyclothiazide. We have shown earlier that cyclothiazide dramatically attenuates the effect of perampanel (ca. 20-fold reduction in activity) and fastens its washout kinetics in isolated CA1 pyramidal neurons (Barygin, 2016). An earlier work with perampanel on cultured hippocampal neurons, in which AMPA receptors were activated by kainate in the absence of cyclothiazide, also demonstrated similar efficiency in preapplication and coapplication protocols (Chen et al., 2014). IEM-1925 of 5 µM inhibited only open channels in a similar experiment on CP-AMPARs of giant striatal interneurons (Figure 9D). Because of the fast kinetics of washout, we were not able to test DNQX in this protocol.

**FIGURE 10:**
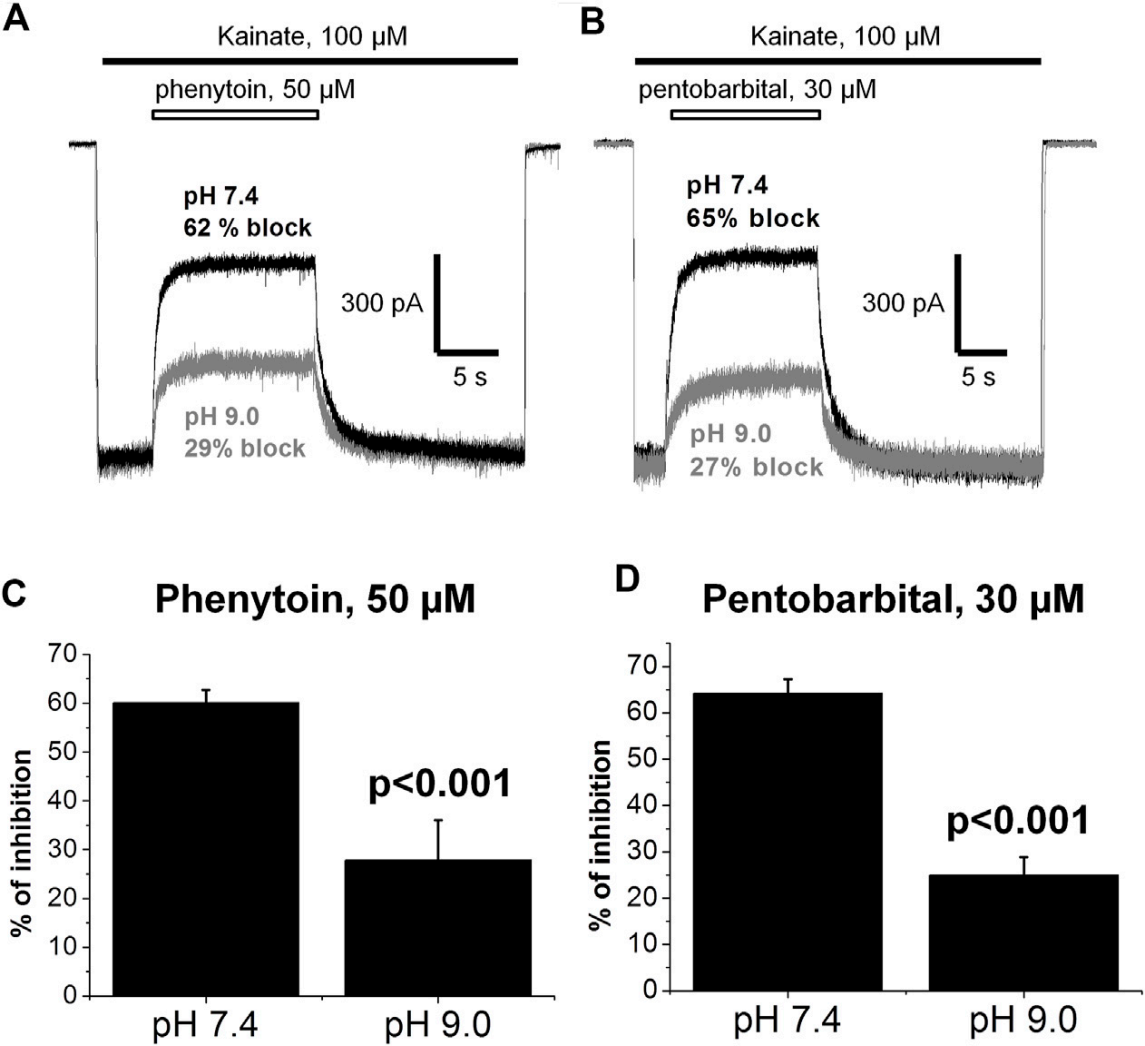
The action of phenytoin and pentobarbital at pH 7.4 and 9.0. **(A,B)** Representative examples of CI-AMPARs inhibition by 50 µM phenytoin **(A)** and 30 µM pentobarbital **(B)** at pH 7.4 (black trace) and 9.0 (gray trace). **(C,D)** Summarized results of CI-AMPARs inhibition by 50 µM phenytoin **(C)** and 30 µM pentobarbital **(D)** at pH 7.4 and 9.0. Phenytoin and pentobarbital were more active at neutral than at more basic pH, which implies that their uncharged forms account for AMPAR inhibition.

In addition, we compared the action of 50 µM phenytoin at two different pHs: 7.4 and 9.0. The pKa value for phenytoin is 8.3 (Agarwal & Blake, 1968). Thus, at pH 7.4, it exists mostly in uncharged form, while at pH 9.0, it is mostly negatively charged. Phenytoin of 50 µM inhibited currents by 60 ± 3% at pH 7.4 and by 28 ± 8% at pH 9.0 (*n* = 6, *p* < 0.001, Figures 10A,C). Such a decrease in phenytoin activity in more basic conditions suggests that the uncharged form of phenytoin produces stronger AMPAR inhibition. Pentobarbital was also more active at neutral than at more basic pH (Figures 10B,D), in line with previous results (Jackson et al., 2003).

## Discussion

In the present work, we have shown for the first time that phenytoin is significantly more active against CI-AMPAR compared to CP-AMPARs. Among known AMPAR antagonists similar selectivity demonstrated pentobarbital (Taverna et al., 1994; Yamakura et al., 1995; Jackson et al., 2003). The action of phenytoin on CI-AMPARs was attenuated in experiments with high agonist concentrations, in the presence of cyclothiazide and at pH 9.0. However, phenytoin was more active in the case of coapplication with agonist compared with preapplication without agonist. Phenytoin demonstrated pronounced trapping when receptors were activated by relatively low kainate concentrations (up to 100 µM), but the trapping was questionable in experiments with higher (500 µM) kainate concentration. This set of features (Table 2) is intriguing because it discriminates phenytoin from three main types of AMPA receptor antagonists: competitive antagonists (e.g., DNQX, CNQX), negative allosteric antagonists (e.g., GYKI-52466, perampanel), and CP-AMPARs selective voltage-dependent channel blockers (e.g., IEM-1925, IEM-1755, argiotoxins, phylantotoxins). Noteworthy, practically the same set of features was shown earlier for pentobarbital (Jackson et al., 2003) and was confirmed in our experiments.

**FIGURE 11:**
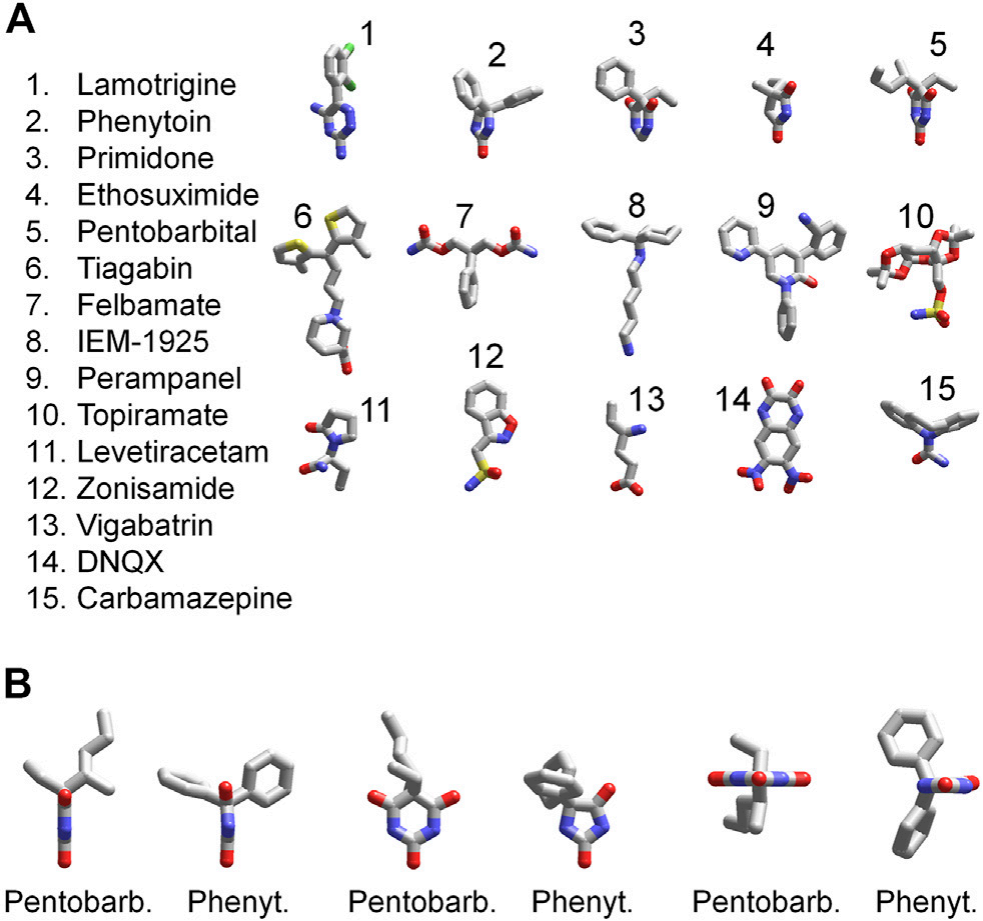
3D structures of compounds. **(A)** Comparison of 3D structures of the compounds. The structures demonstrate large diversity, although compounds 1–5 have certain similarity; they have a heterocycle shown in the bottom and aromatic/hydrophobic moieties shown in the top. **(B)** Comparison of 3D structures of phenytoin and pentobarbital in different orientations. The 3D structures demonstrate close similarity of orientation of functional groups.

The 3D structures of the compounds studied in the present work were calculated by the ZMM software (Figure 11A). They demonstrate huge structural diversity. However, lamotrigine, phenytoin, primidone, ethosuximide, and pentobarbital (compounds 1–5) possess some common motifs. They have a heterocycle (shown at the bottom) and aromatic/hydrophobic moieties (shown at the top). Only phenytoin and pentobarbital demonstrated activity against AMPA receptors, whereas lamotrigine, primidone, ethosuximide, and hydantoin were inactive. This structural comparison indicates that the binding site requirements are rather strong. Both hydrophobic/aromatic moieties and specific mutual disposition of CO and NH groups seen in hydantoin ring and in pyrimidine 2-4-6 trion ring are essential for this type of activity. Figure 11B shows a comparison of phenytoin and pentobarbital in different orientations. In fact, these 3D structures are very similar justifying the common mechanism of action revealed in our experiments.

We have shown that the molecular mechanism of action of phenytoin and pentobarbital on AMPARs is different from that of competitive antagonists, negative allosteric antagonists, and CP-AMPARs selective channel blockers (Table 1). The binding sites for these classical types of AMPAR antagonists are rather well characterized (Tikhonov et al., 2002; Balannik et al., 2005; Yelshanskaya et al., 2016; Twomey et al., 2018; Krintel et al., 2021). But where can the binding site for phenytoin and pentobarbital be situated? For pentobarbital, it has been demonstrated that the single mutation of the Q/R site residue in the GluA2 subunit (R586Q) dramatically decreases the sensitivity (Yamakura et al., 1995) suggesting binding in the central pore at the selectivity filter. Cationic blockers selectively inhibit CP-AMPAR, whereas neutral molecules of pentobarbital and phenytoin can readily bind to the CI-AMPARs containing the Arg residue in the selectivity filter. Although present X-ray and cryo-EM structures seem not precise enough to characterize atomic-scale details of this site unambiguously, it obviously contains hydrophobic central cavity and polar groups serving as proton donors and acceptors (Tikhonov and Zhorov, 2020). Our structure–activity data demonstrate that such features are indeed required to provide inhibitory action of phenytoin and pentobarbital. At a glance, binding in the inner pore region near the selectivity filter is inconsistent with attenuation of inhibitory activity at high kainate concentrations and in the presence of cyclothiazide. However, there are data suggesting that gating rearrangements of AMPA receptor channels involve not only the C-part of the M2 segment but also the selectivity filter (Sobolevsky et al., 2005; Twomey et al., 2017). If it is so, specific drug binding to this site can affect activation properties of the channels and vice versa.

An apparent paradox of the mechanism of action is that phenytoin weakly block closed channels if applied without agonist. However, activation by saturating agonist concentration or enhancing the activation by cyclothiazide also reduces the inhibitory activity of pentobarbital and phenytoin. Although we have no convincing justification for these seemingly controversial data, double-gate mechanism of activation provides a possible explanation. Open conformation of the extracellular gate in the M3 segments is required to free access of external blockers to the binding site, whereas the open state of the gate at the selectivity filter can weaken the drug binding. Since the relationships between the extracellular and the selectivity filter gates are unknown, more detailed explanations seem impractical and premature.

The voltage-gated sodium channels are generally regarded as the main target to explain phenytoin’s activity as an anticonvulsant (Tunnicliff, 1996; Hesselink and Kopsky, 2017a). Affinity of phenytoin to inactivated states of sodium channels is in the range of 7–21 µM (Kuo and Bean, 1994; Lenkowski et al., 2007). Here we have shown for the first time that phenytoin inhibits CI-AMPARs with similar potency. Thus, AMPAR inhibition by phenytoin may contribute to its anticonvulsant and neuroprotective properties, as well as its side effects. While the neuroprotective potential of phenytoin has been evaluated for decades (Stanton and Moskal, 1991; Boehm et al., 1994; Bartollino et al., 2018), the exact molecular mechanisms are not yet clear. It is not yet completely clear even if phenytoin is neuroprotective or neurotoxic (Hesselink and Kopsky, 2017b).

Voltage-gated sodium channels and AMPA receptors are important in keeping proper excitation–inhibition balance in the central nervous system, and the ability of phenytoin to inhibit both of them can underlie its efficiency in case of different types of seizures. Phenytoin is an old drug, and its usage is somewhat limited because of its side effects. Development of new multitarget compounds with the ability to inhibit voltage-gated sodium channels and AMPA receptors seems promising especially for the treatment of drug-resistant epilepsy. Our findings on the structural determinants of action provide a template for further design of selective antagonists of CI-AMPARs.

## Data Availability

The original contributions presented in the study are included in the article/Supplementary Material. Further inquiries can be directed to the corresponding author.

## Abbreviations

AMPA, α-amino-3-hydroxy-5-methyl-4-isoxazolepropionic acid; CP-AMPARs, calcium-permeable AMPA receptors; CI-AMPARs, calcium-impermeable AMPA receptors.
